# Case report: Successful laparoscopic management of early abdominal pregnancy

**DOI:** 10.3389/fmed.2024.1515249

**Published:** 2025-02-04

**Authors:** Haixia Li, Limin Feng, Shengpeng Zhang

**Affiliations:** Department of Gynecology and Obstetrics, Beijing Tiantan Hospital, Capital Medical University, Beijing, China

**Keywords:** abdominal pregnancy, peritoneal cavity, ectopic pregnancy, laparoscopy, intervention

## Abstract

**Background:**

Abdominal pregnancy is characterized by the implantation of the gestational sac within the peritoneal cavity, specifically outside the fallopian tubes, ovaries, and cervix. This exceedingly rare form of ectopic pregnancy accounts for about 1% of all ectopic pregnancies. When rupture occurs, it can result in a life-threatening situation for the patient, necessitating immediate medical intervention.

**Case information:**

In this study, we report a 33-year-old female patient who presented with abdominal pain and was subsequently diagnosed with an early abdominal pregnancy. Laparoscopic intervention revealed gestational tissue and a rupture site located in the pouch of Douglas. The gestational tissue was successfully excised via laparoscopy, and the rupture site was sutured to achieve hemostasis. Postoperative histopathological analysis confirmed the presence of chorionic villi and trophoblast cells.

**Conclusion:**

This case highlights the critical importance of precise diagnosis and laparoscopic intervention in the management of abdominal pregnancy. It is imperative for clinicians to exercise a prudent approach in the diagnosis and treatment of abdominal pregnancy, utilizing a comprehensive assessment that integrates medical history, clinical manifestations, and auxiliary diagnostic tests to enhance the detection and comprehension of this condition.

## Introduction

Abdominal pregnancy represents a rare and severe variant of ectopic pregnancy, defined by the implantation of a fertilized ovum within the abdominal cavity, rather than within the fallopian tubes or ovaries. In cases of abdominal pregnancy, the gestational sac may implant directly onto the peritoneum, mesentery, or omentum, and, in exceptional circumstances, may attach to organs such as the liver or spleen (1–3), it accounts for approximately 1% of all ectopic pregnancies and are associated with a maternal mortality rate of 5.1/1,000 pregnancies (4).

The early symptoms and signs of abdominal pregnancy can often be confused with those of tubal ectopic pregnancies, typically presenting with a history of amenorrhea, lower abdominal pain, and vaginal bleeding, among other symptoms. Notably, only 20–40% of cases are diagnosed preoperatively (1, 5). Given the high risk of life-threatening complications, such as massive hemorrhage and organ damage, timely diagnosis and surgical intervention are critical. This report presents a case of early-stage ruptured abdominal pregnancy that was successfully managed through laparoscopic surgery.

## Case description

A 33-year-old woman, G1P0, presented to the emergency department with a 3-day history of lower abdominal pain. She reported a regular 30-day menstrual cycle, characterized by a 5-day duration of moderate flow and the absence of dysmenorrhea. Nineteen days prior, she experienced vaginal bleeding that aligned with her typical menstrual flow, lasting for 5 days; she did not seek medical attention at that time. Three days ago, following sexual intercourse, she developed intermittent lower abdominal pain, which was not accompanied by vaginal bleeding, anal pressure, dizziness, nausea, or other discomfort. However, as the pain progressively worsened, she sought medical evaluation. She has no significant previous medical history.

## Diagnostic assessment

Physical examination revealed stable vital signs: blood pressure of 113/69 mmHg, pulse of 78 beats per minute, respiratory rate of 20 breaths per minute, and body temperature of 36.5°C. Abdominal examination indicated tenderness without muscle guarding and no evidence of shifting dullness. The gynecological examination showed external genitalia consistent with a married and nulliparous status. The cervix appeared normal, although cervical motion tenderness was noted, along with normal size of uterus and tenderness. There was no rebound tenderness, and no significant abnormalities were detected in the bilateral adnexal regions.

## Laboratory tests

Urine *β*-human chorionic gonadotropin (*β*-hCG) was positive, serum *β*-hCG level of 9256.57 mIU/mL, hemoglobin 118 g/L, white blood cell: 8.35 × 10E^9^/L.

**Figure 1:**
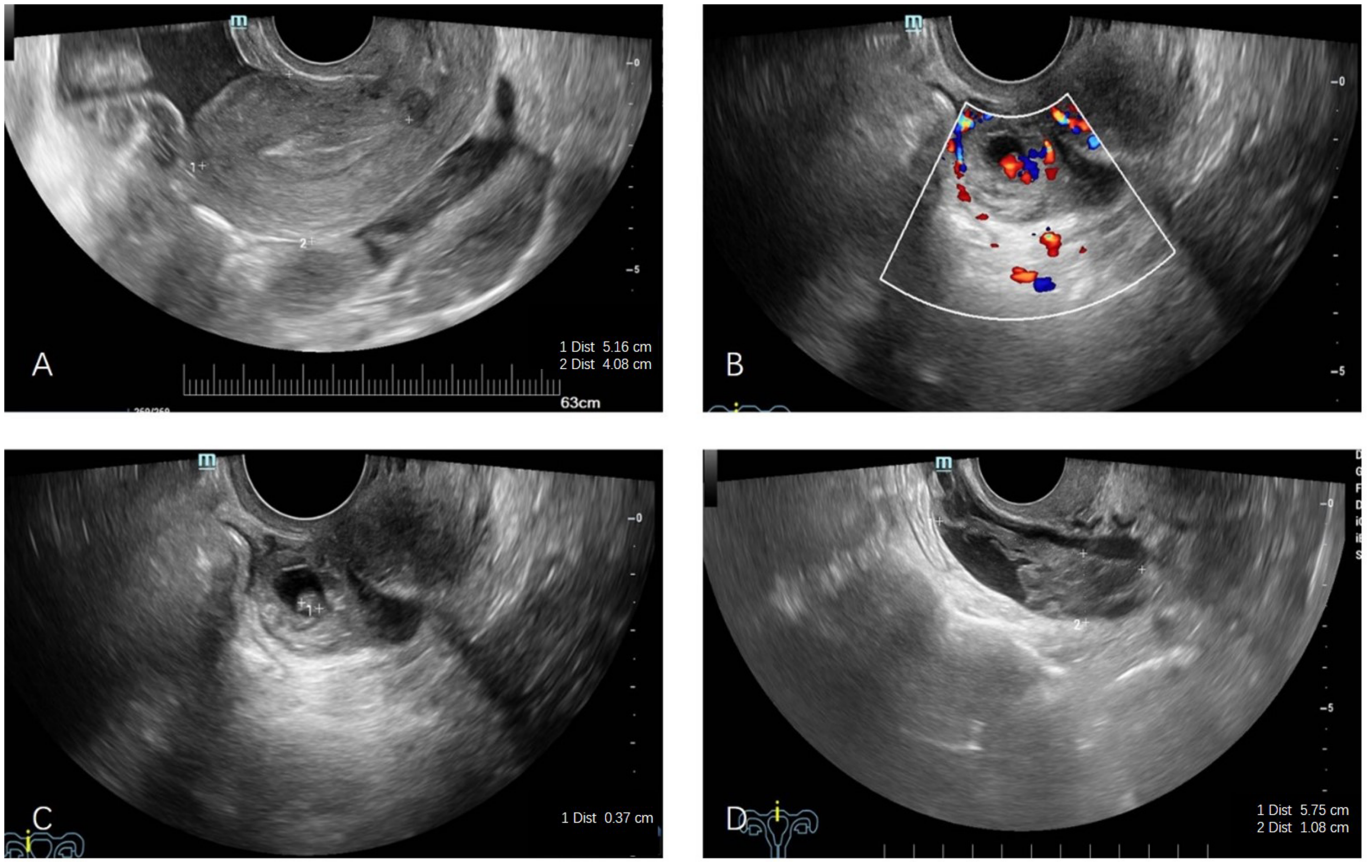
Transvaginal ultrasound examinations. **(A)** No gestational sac was visible within the uterine cavity. **(B)** A gestational sac was identified in the uterorectal pouch, containing a yolk sac approximately 0.4 cm in diameter, with detectable blood flow. **(C)** An embryo bud, measuring approximately 0.4 cm, was observed within the gestational sac in the uterorectal pouch. **(D)** A mixed mass, measuring 5.7 × 1.7 cm, was detected in the pelvic region, with no evidence of blood flow.

Transvaginal ultrasonography revealed a normal uterus (Figure 1A) and identified a mixed mass in the rectouterine pouch, measuring approximately 2.2 × 1.9 cm. This mass exhibited thick peripheral echoes and contained internal small cystic echoes measuring 1.2 × 0.9 cm. Notably, a fetal pole and cardiac activity were observed within the mass, with the fetal pole measuring 0.4 cm in length, and a yolk sac was also detected with a diameter of approximately 0.4 cm (Figures 1B,C). Additionally, a 5.7 × 1.7 cm mixed echogenic mass was noted on the right side of the pelvic floor, characterized by clear boundaries, an irregular shape, uneven internal echoes, and no significant blood flow signal on color Doppler flow imaging (CDFI) (Figure 1D). A small amount of free anechoic fluid was present in the pelvic cavity, with a depth of approximately 4.5 cm and acceptable sound transmission. No significant fluid accumulation was detected in the perihepatic, perinephric, or perisplenic spaces. Abdominal ectopic pregnancy is suspected.

## Treatment

**Figure 2:**
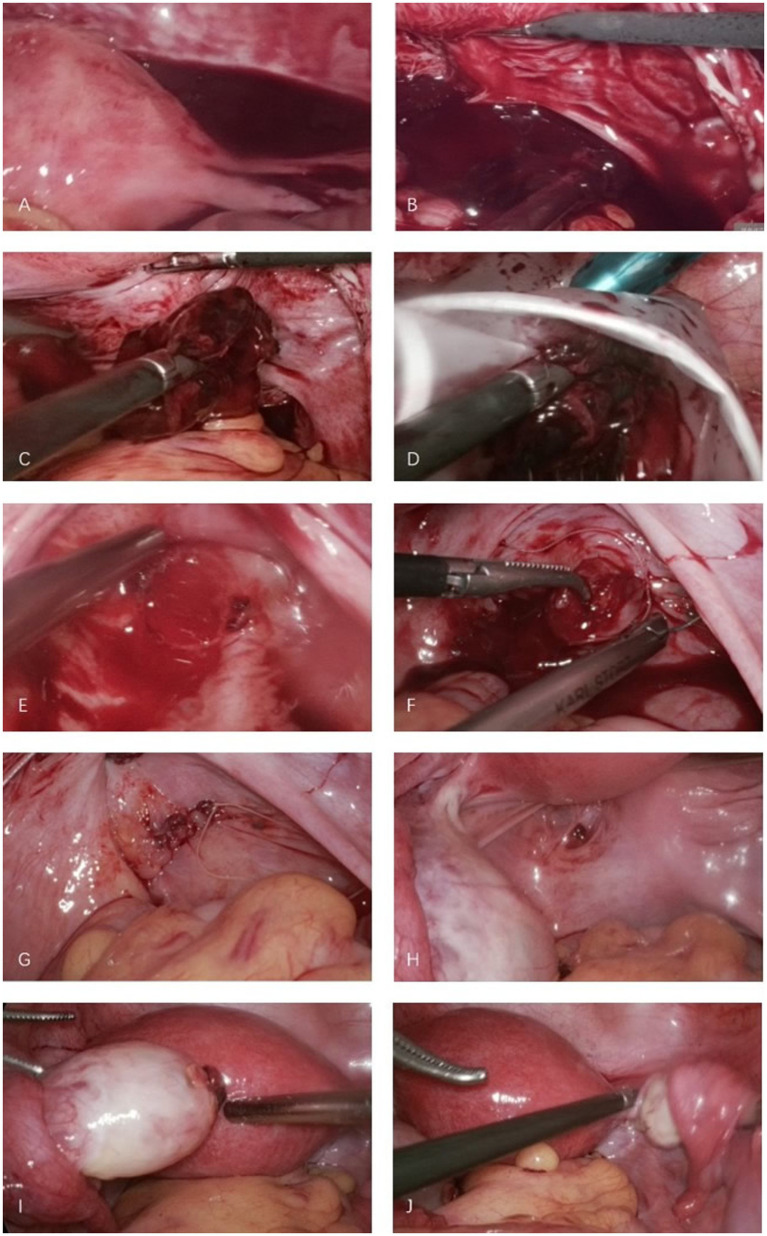
Laparoscopic removal of ectopic pregnancy tissue and repair of uterorectal pouch rupture. **(A)** Hemoperitoneum was noted in the pelvic cavity. **(B)** Hematoma and gestational tissue were identified in the uterorectal pouch (Douglas pouch). **(C)** Laparoscopic graspers were used to remove the hematoma and gestational tissue. **(D)** The hematoma and gestational tissue were placed in an extraction bag and removed out of the abdomen cavity. **(E)** A rupture, approximately 2 cm in diameter, was observed in the uterorectal pouch. **(F)** Continuous suturing of the rupture was performed with 3–0 absorbable sutures to achieve hemostasis. **(G)** No active bleeding was observed upon completion of suturing. **(H)** A patchy “fibrotic retraction” in the peritoneum was noted above the left uterosacral ligament. **(I)** The left ovary appeared mildly enlarged with a visible corpus luteum, while the left fallopian tube was unremarkable. **(J)** The right ovary and fallopian tube appeared normal.

Given the significant risk of hemorrhage associated with ectopic pregnancies, it was imperative to perform blood cross-matching prior to surgery to ensure the availability of blood for transfusion if needed. Laparoscopic exploration was performed immediately, revealing approximately 500 mL of pelvic hematoma. The uterus was of normal size and exhibited a smooth surface. A mass of pregnancy tissue and blood clots, measuring approximately 2.5 × 2 × 1 cm, was located in the rectovaginal pouch, placed in a retrieval bag for removal (Figures 2A–D). Following the aspiration of the pelvic hematoma, a 2 cm tear was identified in the upper portion of the right uterosacral ligament within the rectovaginal pouch, accompanied by evidence of ongoing active bleeding (Figure 2E). The suspected residual products of conception were clamped and excised, with no obvious villous tissue observed upon inspection. Examination of the retrieval bag contents revealed villous and fetal bud tissue, which was subsequently collected and sent for pathological analysis. Hemostasis was achieved through continuous suturing of the superficial wound surface using 3-0 absorbable sutures (Figures 2F,G). A small patchy peritoneal “fibrotic retraction” was noted above the left sacral ligament during exploration (Figure 2H). The left ovary was slightly enlarged, and the left fallopian tube was normal, while the right fallopian tube and ovary appeared normal (Figures 2I,J). No abnormalities were detected during the exploration of the upper abdominal cavity, and no blood transfusion was performed. Intraoperative blood loss (during the procedure of suturing the rupture) was approximately 20 mL.

## Outcome and follow up

**Figure 3:**
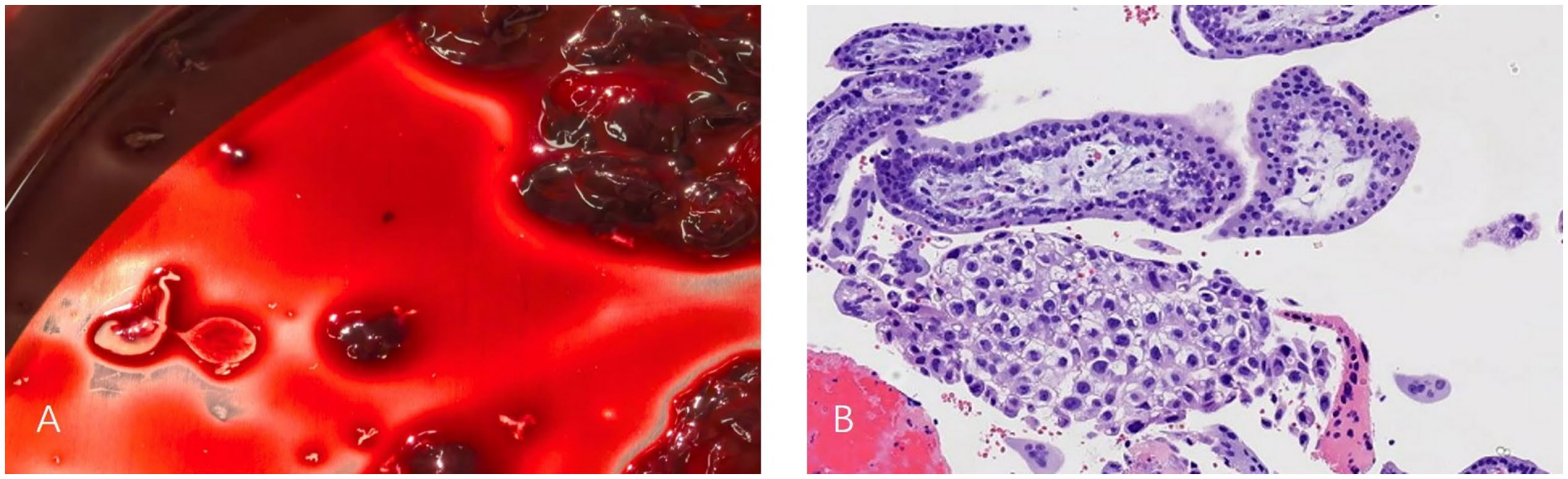
Gross specimen and postoperative histopathological results. **(A)** Visible blood clots and gestational tissue to the naked eye removed from the uterorectal pouch. **(B)** Pathologic examination verified the presence of chorionic villi and trophoblastic cells in the coagulation tissue hematoxylin and eosin staining: ×10.

**Figure 4:**
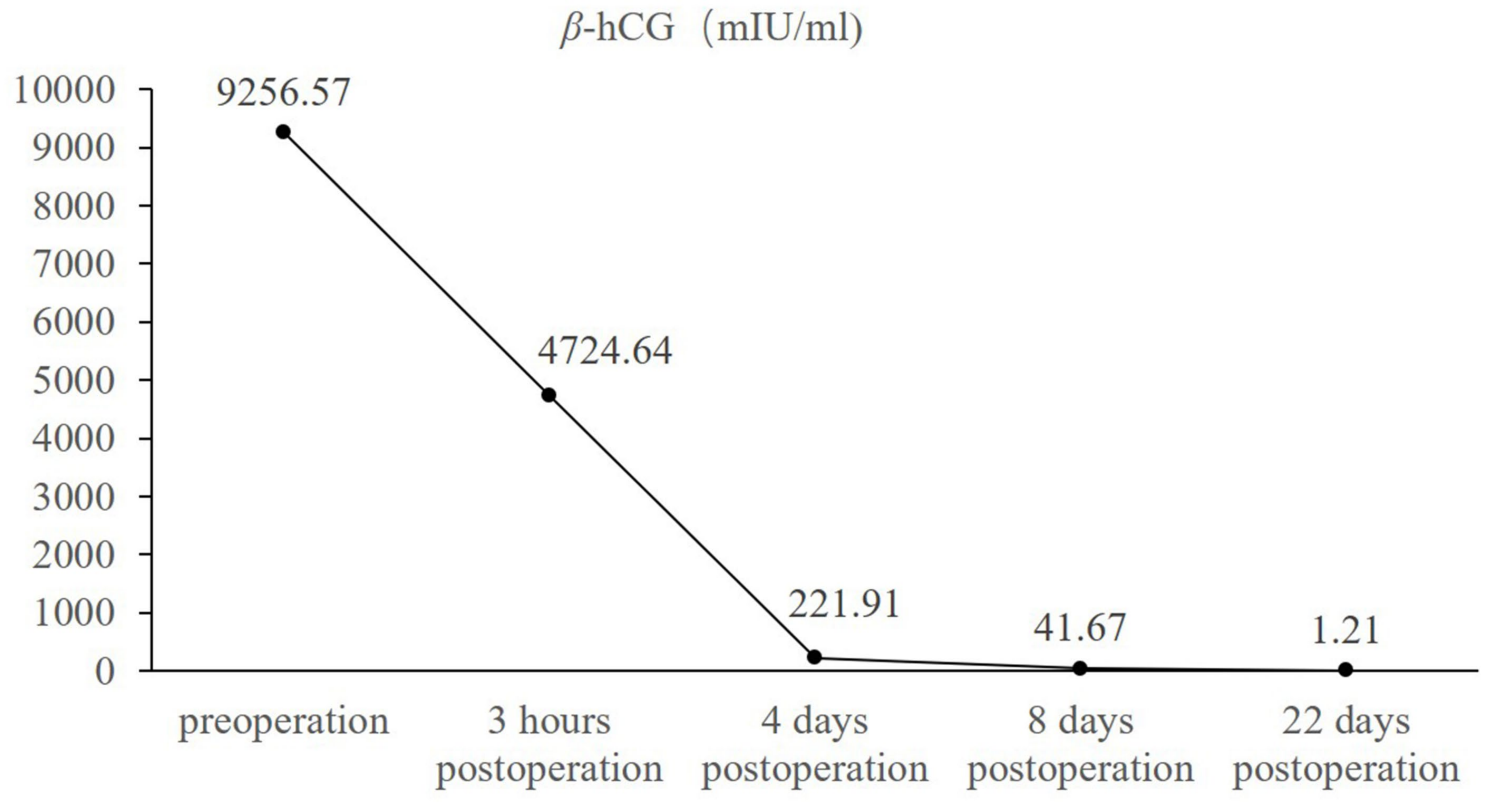
Changes of serum *β*-hCG levels in the patient before and after surgery. *X*-axis: time (before and after surgery), *Y*-axis: level of *β*-hCG (mIU/mL).

The patient had an uneventful postoperative course and was discharged on postoperative day 4. The gross specimen showed visible blood clots and gestational tissue to the naked eye, and histopathological examination of the resected tissue revealed the presence of chorionic villi and trophoblastic cells within the coagulated material (Figures 3A,B). Serial serum *β*-hCG measurements demonstrated a favorable decline, with levels normalizing on postoperative day 22 (1.21 mIU/mL) (Figure 4). A follow-up transvaginal ultrasound performed one month postoperatively showed no abnormalities in the pelvic cavity, uterus or adnexal structures.

## Discussion

Abdominal pregnancy includes primary and secondary types. The diagnostic criteria for primary abdominal pregnancy are as follows: (1) the absence of abnormalities in the bilateral fallopian tubes and ovaries; (2) no evidence of a uterine-peritoneal fistula; and (3) the presence of gestational tissue within the abdominal cavity, with no sign of pregnancy in the uterus, fallopian tubes, or ovaries. Secondary abdominal pregnancy typically occurs after a miscarriage or rupture of a tubal pregnancy and may also arise from ovarian pregnancy or intrauterine pregnancy complicated by uterine defects, such as a scarred uterus or uterine-peritoneal fistula. In these instances, the gestational sac enters the abdominal cavity, implants at a specific site, and continues to develop as an abdominal pregnancy (6, 7).

The etiologies of primary abdominal pregnancy remain poorly understood. High-risk factors associated with abdominal pregnancy include a history of infertility, prior ectopic pregnancy, pelvic surgery, and assisted reproductive techniques such as *in vitro* fertilization and embryo transfer (1, 8, 9). Additionally, Goldman et al. (10) reported three cases potentially linked to the presence of an intrauterine device (IUD), with two cases also involving endometriotic plaques adjacent to the site of pregnancy. Abdominal pregnancy shares common risk factors with other types of ectopic pregnancies, such as intramural, subserosal, and cesarean scar pregnancies. For instance, in the case of parametrial pregnancy, Di Lorenzo et al. (11) discovered pelvic endometriotic lesions during laparoscopic surgery, multiple endometriotic lesions were identified on the peritoneum above the pregnancy mass, and histological examination revealed decidualization of the endometriosis. It was hypothesized that the fertilized ovum implanted in the superficial tissue of the endometriosis and migrated toward the retroperitoneal vascularized area. Adenomyosis, a history of tubal surgery, assisted reproductive technology (ART) involving embryo transfer, and previous interventions in the uterine cavity (such as cesarean sections, which can leave tiny tunnels in the myometrium) may contribute to the risk of intramural pregnancy (12). Prior uterine surgery and procedures involving ART are considered risk factors for subserosal pregnancy (13). Additionally, smoking, number of pregnancies, and deliveries, number of induced abortions, and a history of cesarean section are recognized as high-risk factors for cesarean scar pregnancy (14, 15). Early diagnosis and careful monitoring are essential for managing these potentially life-threatening conditions.

In this report, we describe a case of primary abdominal pregnancy successfully managed through laparoscopic surgery. The patient had a history of regular menstrual cycles and dysmenorrhea-free menses. During laparoscopic inspection, a peritoneal “fibrotic retraction” approximately 1 cm in diameter was identified on the lateral aspect of the left uterosacral ligament. No typical blue-black nodules or other visible signs of endometriosis were present, and the etiology is uncertain.

The clinical manifestations of early abdominal pregnancy are often nonspecific, complicating early diagnosis. Patients may present with vague abdominal or gastrointestinal symptoms, which further delays timely recognition. Despite routine prenatal care and ultrasound evaluations, many cases remain undiagnosed until surgical intervention (16, 17). Transvaginal ultrasound provides significant advantages due to its proximity to the lesion site, enabling higher-resolution imaging and increased sensitivity to blood flow signals. This approach is particularly adept at detecting subtle lesions. By positioning the transducer in the vaginal fornix, the procedure minimizes interference from intestinal gas or abdominal wall adiposity, thereby optimizing image clarity and diagnostic accuracy. Given to its non-invasive, painless, and accessible nature, ultrasound remains the first-line imaging modality for diagnosing ectopic pregnancy.

However, in the context of abdominal pregnancy, variability in physician expertise can lead to a higher rate of misdiagnosis via ultrasound. Diagnostic sensitivity increases when ultrasound is used in conjunction with serum *β*-hCG levels. If ultrasound does not yield a definitive diagnosis of abdominal pregnancy, magnetic resonance imaging (MRI) should be considered. MRI provides additional, detailed insights, particularly in evaluating placental invasion into abdominal and pelvic structures. Its use is recommended in cases where a precise diagnosis is necessary and when no contraindications are present (18).

In the reported case, the patient presented with lower abdominal pain following sexual intercourse, while maintaining regular menstrual cycles. Ultrasound examination identified a mildly enlarged left ovary without accompanying vaginal bleeding, which could have resulted in a misdiagnosis of corpus luteum rupture or other etiologies of acute abdomen. The ultrasound further revealed the presence of a gestational sac in the rectouterine pouch and pelvic hemorrhage, and this finding, corroborated by both urine and serum *β*-hCG tests, confirmed the diagnosis of an abdominal pregnancy.

Abdominal pregnancy presents significant diagnostic challenges, particularly before rupture and hemorrhage occur. Even when clinical symptoms such as rupture, bleeding, and abdominal pain are present, the condition is often misdiagnosed as another pathology. The gestational sac may rupture, allowing the embryo to invade adjacent maternal organs and major blood vessels, potentially leading to catastrophic hemorrhage or organ rupture. This can precipitate hemorrhagic shock, posing an immediate and life-threatening risk to the patient. Patients with abdominal pregnancy present with severe signs of internal bleeding and may already be in shock before seeking medical care.

Surgical intervention is often necessary, with two main approaches: laparotomy and laparoscopic surgery. Laparoscopic surgery is generally preferred because it is minimally invasive, allows for quicker postoperative recovery, and provides better visualization. However, in cases of severe intra-abdominal bleeding or hemodynamic instability, laparotomy may be required. Tanase et al. (19) have reported a case of primary omental pregnancy in a patient with a history of myomectomy and left ovarian cyst enucleation, complicated by severe pelvic adhesions. After adhesiolysis, no pregnancy tissue was found in the pelvis; however, ectopic tissue was discovered in the omentum during laparoscopic exploration. When neither fallopian tube nor ovarian pregnancy is found during laparoscopic or open surgery, it is crucial to thoroughly examine all surrounding structures, including the peritoneal surfaces, omentum, bowel, ureters, cervix, and vagina. This thorough examination is essential to minimize postoperative complications (19, 20).

Laparoscopic examination in this patient revealed that the ruptured gestational tissue was located in the rectouterine pouch, without involvement of the lower segment of the posterior uterine wall. A superficial rupture was sutured at the rectouterine pouch. In cases of abdominal pregnancy involving the rectouterine pouch, it is critical to clearly assess the depth of gestational tissue invasion to prevent damage to the intestines during resection. Pathological examination of the excised abdominal contents confirmed the presence of chorionic villi and fetal tissue. Postoperatively, the patient’s serum *β*-hCG levels showed a significant decrease, returning to normal within about 3 weeks.

Ectopic pregnancy may be managed through surgical intervention or pharmacological treatment. Surgical management is typically indicated in cases where the patient is hemodynamically unstable, presents with a ruptured ectopic pregnancy, experiences intraperitoneal bleeding, or when medical management is contraindicated. At present, there is no definitive consensus regarding the routine use of methotrexate (MTX) for the treatment of abdominal pregnancy, and the available evidence is limited. Reports on the conservative management of abdominal pregnancy using medication are relatively rare, with the majority being individual case studies (21). This approach is primarily reserved for selected patients in stable condition, without significant signs of intra-abdominal bleeding, or as a postoperative adjunct therapy (22). Close monitoring of the patient’s vital signs is essential during conservative drug treatment.

In summary, early primary abdominal pregnancy often presents with nonspecific symptoms, making diagnosis challenging and increasing the risk of missed or misdiagnosed cases. Clinicians should exercise caution during diagnosis and treatment, relying on medical history, clinical presentation, and auxiliary tests (such as urine *β*-hCG, serum *β*-hCG, and transvaginal ultrasound). Once rupture occurs, it poses a significant threat to the patient’s life. Prompt surgical intervention is critical for improving outcomes, with laparoscopic surgery being the preferred approach for both diagnosis and treatment.

## Data Availability

The original contributions presented in the study are included in the article/supplementary material, further inquiries can be directed to the corresponding author.
